# Sertraline induces DNA damage and cellular toxicity in Drosophila that can be ameliorated by antioxidants

**DOI:** 10.1038/s41598-020-61362-y

**Published:** 2020-03-11

**Authors:** Arpita Jajoo, Catherine Donlon, Sarah Shnayder, Michael Levin, Mitch McVey

**Affiliations:** 1 Department of Biology, Tufts University, Medford, MA USA; 2 Allen Discovery Center at Tufts University, Medford, MA USA

**Keywords:** Mechanisms of disease, Drosophila

## Abstract

Sertraline hydrochloride is a commonly prescribed antidepressant medication that acts by amplifying serotonin signaling. Numerous studies have suggested that children of women  taking sertraline during pregnancy have an increased risk of developmental defects. Resolving the degree of risk for human fetuses requires comprehensive knowledge of the pathways affected by this drug. We utilized a *Drosophila melanogaster* model system to assess the effects of sertraline throughout development. Ingestion of sertraline by females did not affect their fecundity or embryogenesis in their progeny. However, larvae that consumed sertraline experienced delayed developmental progression and reduced survival at all stages of development. Genetic experiments showed that these effects were mostly independent of aberrant extracellular serotonin levels. Using an *ex vivo* imaginal disc culture system, we showed that mitotically active sertraline-treated tissues accumulate DNA double-strand breaks and undergo apoptosis at increased frequencies. Remarkably, the sertraline-induced genotoxicity was partially rescued by co-incubation with ascorbic acid, suggesting that sertraline induces oxidative DNA damage. These findings may have implications for the biomedicine of sertraline-induced birth defects.

## Introduction

Serotonin (5-hydroxytryptamine, or 5-HT) is a neurotransmitter that regulates several behaviors in the metazoan, such as mood, social behavior, sleep, and food intake^1^. It is also important for embryogenesis, and contributes to oocyte maturation, vascular remodeling, neural crest migration, craniofacial and limb development, left-right asymmetry, cytoskeletal structure, blastomere adhesion, neural patterning, and gastrulation ^2–6^. Upon release by a presynaptic neuron into the synaptic cleft, serotonin binds 5-HT receptors on the postsynaptic neuron and initiates downstream signaling. Signaling is attenuated via reuptake from the synapse into the presynaptic neuron by the serotonin transporter (SERT) or via degradation by monoamine oxidase. Imbalanced serotonin levels are associated with several mental disorders, such as anxiety, depression, post-traumatic stress disorder, and obsessive-compulsive disorder, and this reuptake mechanism is often targeted to restore serotonin levels in patients with these disorders. Selective Serotonin Reuptake Inhibitors (SSRIs) block SERT, thus leading to higher serotonin concentration and longer duration of action in the synapse. Disorders of serotonergic signaling can also contribute to metastatic cell behavior^7–9^.

Sertraline hydrochloride, henceforth referred to as “sertraline” and marketed under the brand name Zoloft®, is a commonly prescribed SSRI for numerous disorders^10^. Preclinical trials with sertraline in laboratory animals reported no evidence that sertraline is genotoxic^11^ and a small study using peripheral lymphocytes from human patients found no significant effects of sertraline on numbers of chromosome aberrations^12^. Despite the laboratory studies that used sertraline to perturb ion channels and a number of cellular signaling pathways ^13–21^, sertraline currently has Category C designation by the FDA. This has led to its becoming one of the most frequently used SSRIs by pregnant women^22^. An analysis performed in 2015 failed to find significant evidence for harmful effects associated with sertraline use during pregnancy^23^. However, other studies have demonstrated that sertraline usage during pregnancy is linked to adverse short- and long-term effects in children^24^, including atrial septal defects^25^, craniosynostosis^26^, increased risk of omphalocele^27^, developmental delays and increased risk of autism in males^28^, increased risk of clubfoot^29^, elevated risk of persistent pulmonary hypertension^30^, decreased birth weight^31^, and preterm births^32^. These structural phenotypes are in addition to a rich literature discussing behavioral outcomes of SSRI exposure during gestation ^33,34^.

The phenomenon of withdrawal (neonatal abstinence syndrome) reveals the profound effects that exposure to SSRIs has on embryos^35^. Similar to the developmental defects described above, the observed withdrawal effects present stochastically in the human population, resulting in debate over the relative risks of exposure. Importantly, regulative developmental mechanisms and physiological as well as genetic heterogeneity of both mother and fetus result in a variability of phenotypes across the population, as has been seen in developmental biology studies in animal model systems^36^. Because different embryos may succumb to or resist perturbation of specific steps in development, it is critical to comprehensively characterize the means by which sertraline (and other SSRIs) could impact embryogenesis. Disruption of the known developmental roles of serotonergic signaling has been suggested to be one of the routes of defects in some individuals^37–39^. However, it is also possible that sertraline results in genetic, epigenetic, or other cellular abnormalities that affect developmental patterning.

Ethical considerations prevent randomized controlled clinical trials in humans to determine sertraline’s embryonic effects and risk profile. To circumvent these challenges, model organisms have been utilized to investigate the effects of sertraline exposure at both cellular and organismal levels. Studies with *Xenopus laevis* showed that exposure of tadpoles to sertraline (0.1–10 μg/L) resulted in developmental toxicity^40^. Furthermore, mice exposed to low levels (10 μM) of sertraline develop craniofacial abnormalities, due to interference with serotonin signaling important for normal craniofacial development^15^.

Drosophila is one of the premiere organisms for studying developmental processes, and is a powerful model for discovery of mechanisms of relevance to human medicine including cancer, neurobiology, and left-right asymmetry^41–43^, all of which are areas where SSRIs have been strongly implicated. The genome of *Drosophila melanogaster* contains homologs of ~75% of human disease-related genes^44^. Drosophila utilize serotonin as a neurotransmitter in the nervous system and as a small molecule in early development^45,46^ and have serotonin receptor homologs. Although no monoamine oxidase has been described in *Drosophila*, their genome does encode a single serotonin reuptake transporter, named SerT^47^. Thus, we took advantage of this powerful developmental model to search for novel mechanisms for sertraline action in development, specifically focusing on its ability to disrupt genetic integrity.

Only one study has been published investigating the genotoxicity of sertraline in Drosophila. Using an assay called the somatic mutation and recombination test (SMART), the authors showed that citralopram, another SSRI, caused dose-dependent genotoxicity, but the results with sertraline were inconclusive^48^.

To further investigate possible genotoxic effects of sertraline in Drosophila and to identify possible causes for the sertraline-related birth defects in humans, we performed a series of experiments at larval and adult stages to determin the impact of sertraline on development and survival. In addition, we incubated larval wing disc tissues with sertraline to investigate changes in cellular markers related to cell death and DNA damage. While exposure of female adult flies to sertraline had no effects on egg laying or hatching, larval exposure led to delays in developmental progression and to impaired viability. Mechanistically, sertraline induced oxidative damage of DNA, which caused cellular apoptosis in tissues necessary for proper development. Strikingly, the cellular damage induced by sertraline could be rescued by antioxidant supplementation. These results establish sertraline as detrimental to fly development, elucidate a new mechanism of its developmental toxicity, and for the first time, provide a potential avenue for mitigation of its adverse effects on embryogenesis that is highly compatible with human patient use.

## Results

### Sertraline-treated adult females exhibit no difference in fecundity and embryo hatching frequency

**Figure 1:**
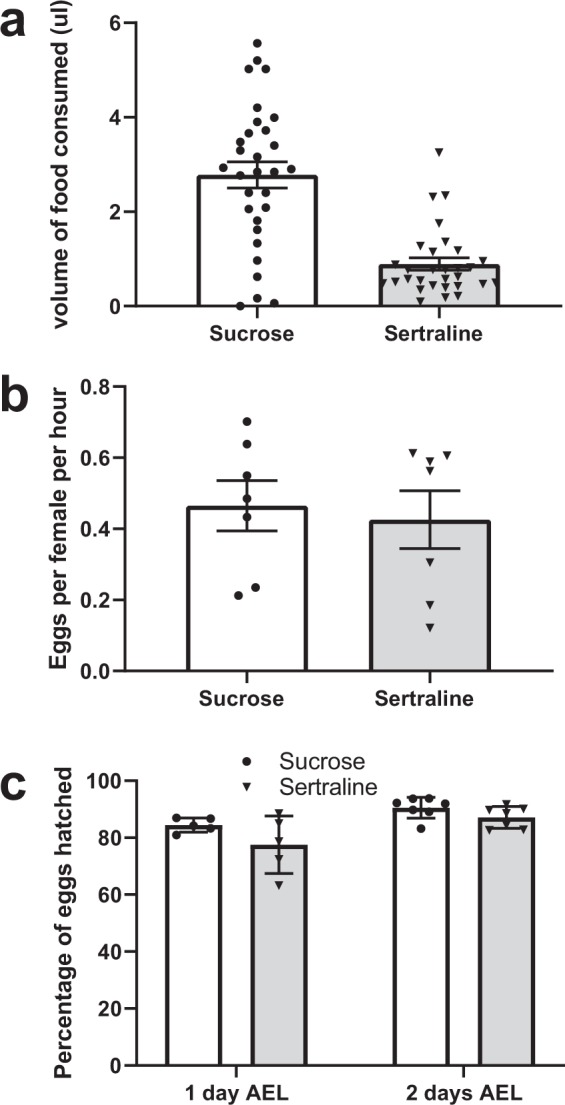
Sertraline consumption does not impact fecundity or egg hatching frequency in Drosophila. (**a**) Amount of liquid consumed by 8 adult female flies during 8 hours from a single capillary tube in a CAFE apparatus. Each tube contained either 5% sucrose or 250 μg/ml sertraline in 5% sucrose. N = 30, error bars represent SEM. p < 0.0001, unpaired two-tailed t test. (**b**) Egg laying calculated as the number of eggs laid by one female per hour after treatment with either sucrose or 250 μg/mL sucrose + sertraline solution. N = 7, error bars represent SEM. p = 0.72, unpaired two-tailed t-test. (**c**) Percentage of eggs hatched one day and two days after egg laying (AEL) by mothers fed either sucrose solution or 250 μg/ml sertraline solution. N = 5–7 replicates for each condition, error bars represent SEM. p = 0.18 after 1 day and p = 0.11 after 2 days, unpaired two-tailed t-test.

We began by investigating the effects of sertraline ingestion on the quantity and quality of eggs laid by adult females. Cohorts of eight females 3–4 days of age were fed using capillary feeders in a modified CAFE apparatus^49^. Each apparatus had capillaries filled with either a 5% sucrose solution or 5% sucrose with 250 μg/mL of sertraline, a non-toxic concentration for adult Drosophila^48^. Although the flies consumed less liquid in the apparatus that contained sertraline solution, we were successful in exposing the adult females to the drug via this method (Fig. 1a).

Females that were fed only sucrose or sertraline were then placed into a cage with equal numbers of male flies and the number of eggs laid by the females in an 18-hour period was counted. Compared to sucrose, we found no significant difference in fecundity of female flies that drank sertraline-containing solutions (Fig. 1b). On average, female flies fed plain sucrose laid 1 egg every 2.15 hours, while females fed sucrose with sertraline laid 1 egg every 2.34 hours (p = 0.72).

In addition, there was no significant difference in the number of eggs that hatched between the sertraline-fed and control flies (Fig. 1c). One- and two-day hatching frequencies for eggs laid by control females were 84% and 91%, compared to frequencies of 78% and 87% for sertraline-treated females (p = 0.11). To determine if sertraline fed to adult females affected the development of their progeny, we allowed larvae from these eggs to develop into adulthood. Of more than five hundred progeny examined, no obvious morphological or phenotypic differences were noted for progeny from the sertraline-treated females. Thus, while we cannot rule out subtle effects on development, sertraline-fed females do not appear to produce offspring with obvious morphological defects.

### Sertraline-treated larvae display delayed development and decreased survival

Unlike human development, which takes place *in utero* and exposes the fetus to sertraline ingested by its mother, Drosophila embryonic development takes place outside of the mother in an egg that is protected from the environment through an impermeable vitelline membrane and chorion. Larvae which hatch from the egg continue development, paralleling the various stages of organogenesis and remodeling that occur in embryos of all species. After hatching, Drosophila larvae consume food and grow through three instar stages, each lasting approximately 24 hours. At the end of the third instar stage, they cease eating and enter the wandering third instar stage, which lasts for several hours and is followed by a cessation of movement and pupariation. During the pupal stage, which lasts approximately 3–4 days, they undergo metamorphosis and then emerge as adult flies.

We wanted to test whether direct exposure to sertraline might affect Drosophila during its various developmental stages. Repeated attempts to introduce sertraline directly into eggs drastically reduced the viability of the embryos. Therefore, we turned to an established larval feeding protocol, in which larvae consume sertraline-containing food throughout their development^48^. First instar larvae derived from an *Oregon-R* stock of flies were placed onto food containing different concentrations of sertraline and their rate of development to various developmental stages was quantified and compared to larvae placed in vehicle-containing food (see Methods).

**Figure 2:**
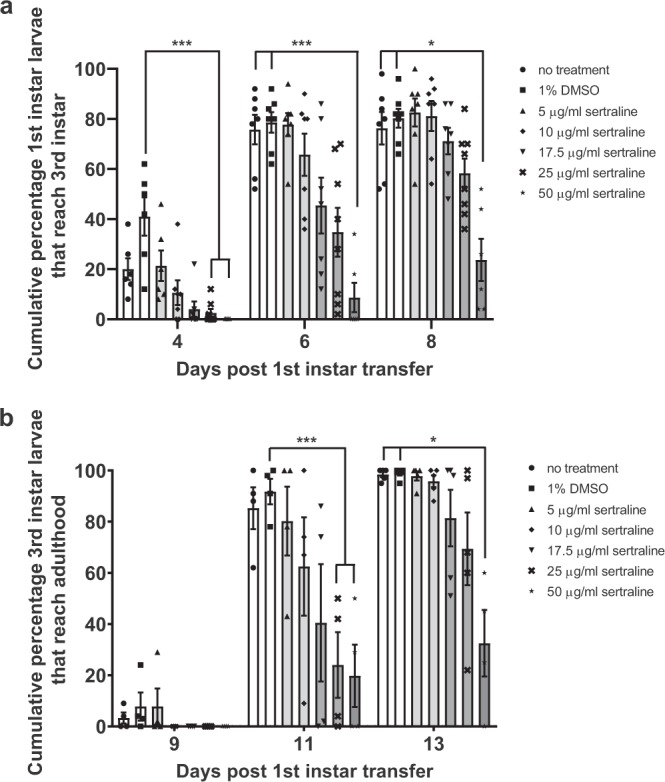
Sertraline delays Drosophila development and reduces survival frequency. (**a**) Cumulative percentage of wandering third instar larvae following placement of 20–25 Oregon-R first instar larvae into food containing various concentrations of sertraline at day 0. (**b**) Cumulative percentage of surviving third instar larvae that developed to adulthood following placement of first instar larvae into food containing various concentrations of sertraline at day 0. Each trial lasted for at least 15 days, with no additional changes observed after day 13. N = 4–7 trials for each treatment, error bars represent SEM. *p < 0.05 and ***p < 0.001 (one-way ANOVA with Bonferroni correction).

We chose the transition to the wandering third instar stage as our first developmental time point. We observed a dose-dependent effect for development to wandering third instar larvae, with significant delays observed for the two highest concentrations of sertraline (Fig. 2a). In addition, there was an overall decrease in the survival in larvae treated with sertraline, with only 33% of first instar larvae treated with the highest sertraline concentration surviving to third instar stage, compared to 80% for the controls.

A similar trend was observed for development from third instar larvae to adulthood (Fig. 2b). For this analysis, we counted the number of adults that eclosed from their pupal cases on successive days and calculated the percentage of adults relative to the number of total third instar larvae that we obtained for each treatment. The developmental delay to the mature adult stage for the highest concentrations of sertraline was similar to the delay seen for the transition from first to third instar larvae, suggesting that the overall delay in development occurred during the larval stages, when the sertraline-containing food was being consumed. However, we also observed a further decrease in survival between the third instar and adult stages for the highest concentrations of sertraline. In the control experiments, over 98% of the wandering third instar larvae survived to adulthood, while only 33% of the third instar larvae raised in the 50 μg/mL sertraline-containing food successfully pupated and eclosed as adults. Therefore, sertraline continues to affect Drosophila viability beyond the larval period when it is being actively ingested.

### Sertraline affects Drosophila development independently of serotonin-mediated effects

The developmental delays and decreased survival caused by exposure to sertraline could be caused by changes in serotonin levels due to inhibition of the serotonin reuptake mechanism or by genotoxicity of sertraline itself. To distinguish between these two possibilities, we repeated the larval feeding experiments with flies lacking the sole serotonin reuptake transporter (hereafter referred to as *SerT* knockout or *SerT −/−* flies). We chose a sertraline concentration of 10 μg/ml, where we previously observed moderate effects on developmental timing, reasoning that in the *SerT* knockout flies, any additional developmental delays phenotypes observed in the presence of sertraline treatment could be attributed to genotoxic effects of sertraline itself, rather than its effects on the serotonin transporter. As a control, we measured developmental progression in isogenic flies with an intact *SerT* gene (see Methods).

**Figure 3:**
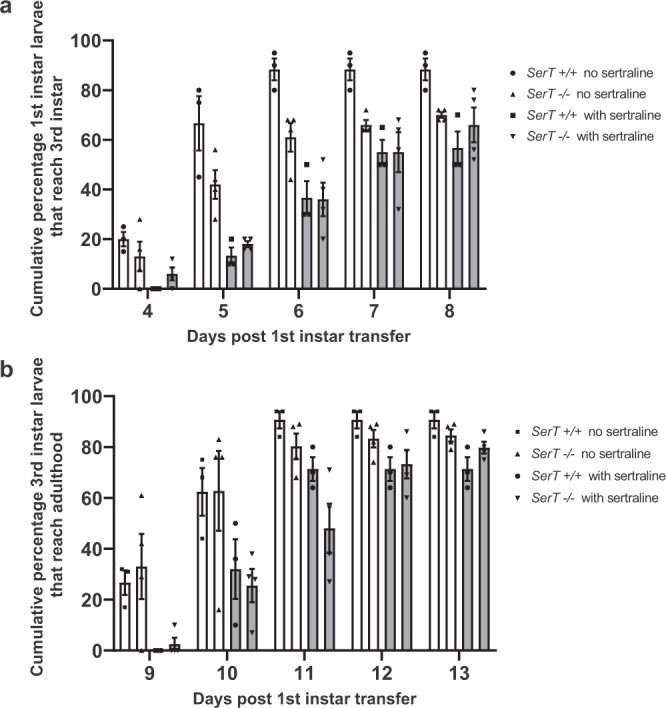
The effects of sertraline on development are mostly independent of its effects on serotonin signaling. (**a**) Cumulative percentage of wandering third instar larvae following placement of 20–25 first instar larvae into food containing 10 μg/ml sertraline at day 0. (**b**) Cumulative percentage of surviving third instar larvae that developed to adulthood following placement of first instar larvae into food containing 10 μg/ml sertraline at day 0. Each trial lasted for at least 15 days, with no additional changes observed after day 13. *SerT −/−* flies lack the SerT serotonin reuptake channel and *SerT* +*/+* flies possess a wild-type copy of the *SerT* gene in an isogenic background. N = 4 replicates for *SerT −/−* and 3 replicates for *SerT* +*/+*, error bars represent SEM.

As observed with the *Oregon-R* flies, there was approximately a 1-2 day developmental delay from first-instar to wandering third instar larvae in the presence of 10 μg/mL sertraline, for both the *SerT* knockout and *SerT* wild-type stocks (Fig. 3a). Similarly, sertraline-treated flies from both stocks exhibited a delay in development between the wandering third instar and adult stages (Fig. 3b). While the flies lacking SerT did have a slightly delayed transition from first to third instar larvae in the absence of sertraline, this developmental delay was ameliorated by the time the flies reached adulthood. In addition, we observed no increase in sertraline-mediated lethality in the *SerT* knockout larvae compared to *SerT* wild-type larvae. From these data, we conclude that while a portion of the larval developmental delays caused by sertraline ingestion may be due to changes in serotonin levels, most of the sertraline-induced delays and lethality is independent of its effects on serotonin signaling. Thus, we next examined its genotoxic potential.

### Sertraline induces apoptosis and DNA damage in wing imaginal discs

**Figure 4:**
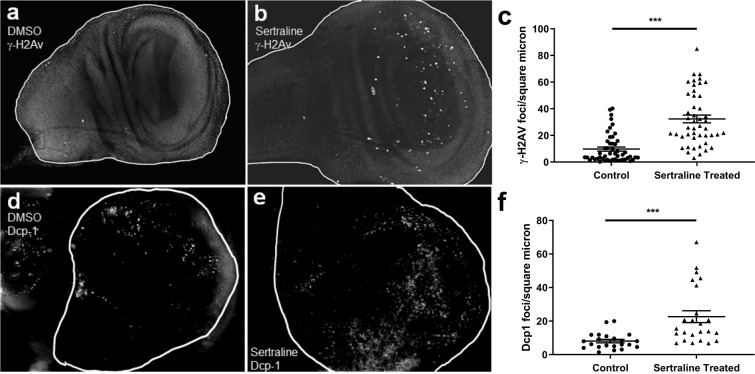
Sertraline causes accumulation of DNA double-strand breaks and cell death in wing imaginal discs. (**a** and **b**) Wing imaginal discs dissected from *SerT* −/− wandering third instar larvae were treated for 5 hours with DMSO or 10 μg/ml sertraline and stained with antibodies that recognize phosphorylated γ-H2Av. (**c**) Quantification of γ-H2Av foci per square micron in control and sertraline treated discs. γ-H2Av foci were counted and normalized to the area of each wing disc, measured from DAPI-stained images. N = 50 discs for control and 47 for sertraline treatment, error bars represent SEM. ***p < 0.001 by unpaired t test with Welch’s correction. (**d** and **e**) Wing imaginal discs dissected from *SerT* −/− wandering third instar larvae, treated with DMSO or 10 μg/ml sertraline, and stained with antibodies against cleaved Dcp-1. (**f**) Quantification of the number of Dcp-1 foci per square micron in control and sertraline-treated discs. Dcp-1 foci were counted and normalized to the area of each wing disc, measured from DAPI-stained images. N = 22 discs for control and 24 for sertraline treatment, error bars represent SEM. ***p = 0.0005 by unpaired t test with Welch’s correction.

In Drosophila, larval imaginal disc tissues become adult structures, such as eyes, wings, and antennae, following metamorphosis. The induction of DNA damage in imaginal disc tissues results in cell death and developmental delays^50–52^. We hypothesized that sertraline might induce DNA double-strand breaks, the accumulation of which could result in apoptosis in larval imaginal discs, developmental delays, and organismal lethality. To test this hypothesis, we measured levels of DNA damage following sertraline treatment in cultured wing imaginal discs of *SerT* knockout third instar larvae. We have previously shown that cells in cultured imaginal discs can survive and continue to divide for at least five hours^53^. In Drosophila, phosphorylation of the histone H2A variant on serine-137 (γ-H2Av) occurs upon double-strand break formation and the number of γ-H2Av foci is roughly proportional to the number of breaks ^54,55^. Strikingly, a five-hour incubation of wing imaginal discs with 10 μg/mL sertraline resulted in an average three-fold increase in the number of γ-H2Av foci (Fig. 4a,b). Untreated wing discs displayed 10 ± 11 γ-H2Av foci/micron^2^, while sertraline treated discs had an average of 32 ± 20 foci/micron^2^ (Fig. 4c), indicating that sertraline treatment induces higher levels of DNA damage in proliferating discs.

To determine whether the elevated levels of DNA damage also lead to increased cell death, we monitored apoptosis via staining of cleaved death caspase-1 (Dcp-1), which is an effector caspase that promotes degradation of target proteins during apoptosis^56,57^. Wing discs cultured in the absence of sertraline displayed expected patterns of apoptosis, with Dcp-1 foci clustered mostly towards the top of the discs (Fig. 4d) and little in the middle folded “frown” region^58^. Although the number of apoptotic cells in sertraline treated discs was more variable, on average the treated discs had three-fold greater numbers of Dcp-1 foci compared to the control discs. Thus, exposure of tissues to sertraline for just a few hours can induce DNA damage in the form of double-strand breaks and lead to excessive cell death that correlates with a delay in organismal development.

### Antioxidant supplementation can ameliorate DNA damage induced by sertraline exposure

Beyond its extracellular interactions with the serotonin transporter, sertraline can freely diffuse into cells, and its movement is accelerated by vacuolar proton ATPases^59^. Although sertraline’s interactions with genetic material have not been investigated *in vivo*, sertraline does have affinity for the minor groove of DNA^60^, indicating its potential to interact with an organism’s genetic material once inside the cell. Interestingly, serotonin can act as a pro-oxidant of DNA^61^ and induce double strand breaks in the presence of copper ions^62^. Thus, we hypothesized that sertraline, or one of its metabolic breakdown products, might induce oxidative DNA damage that could eventually lead to the formation of double-strand breaks.

**Figure 5:**
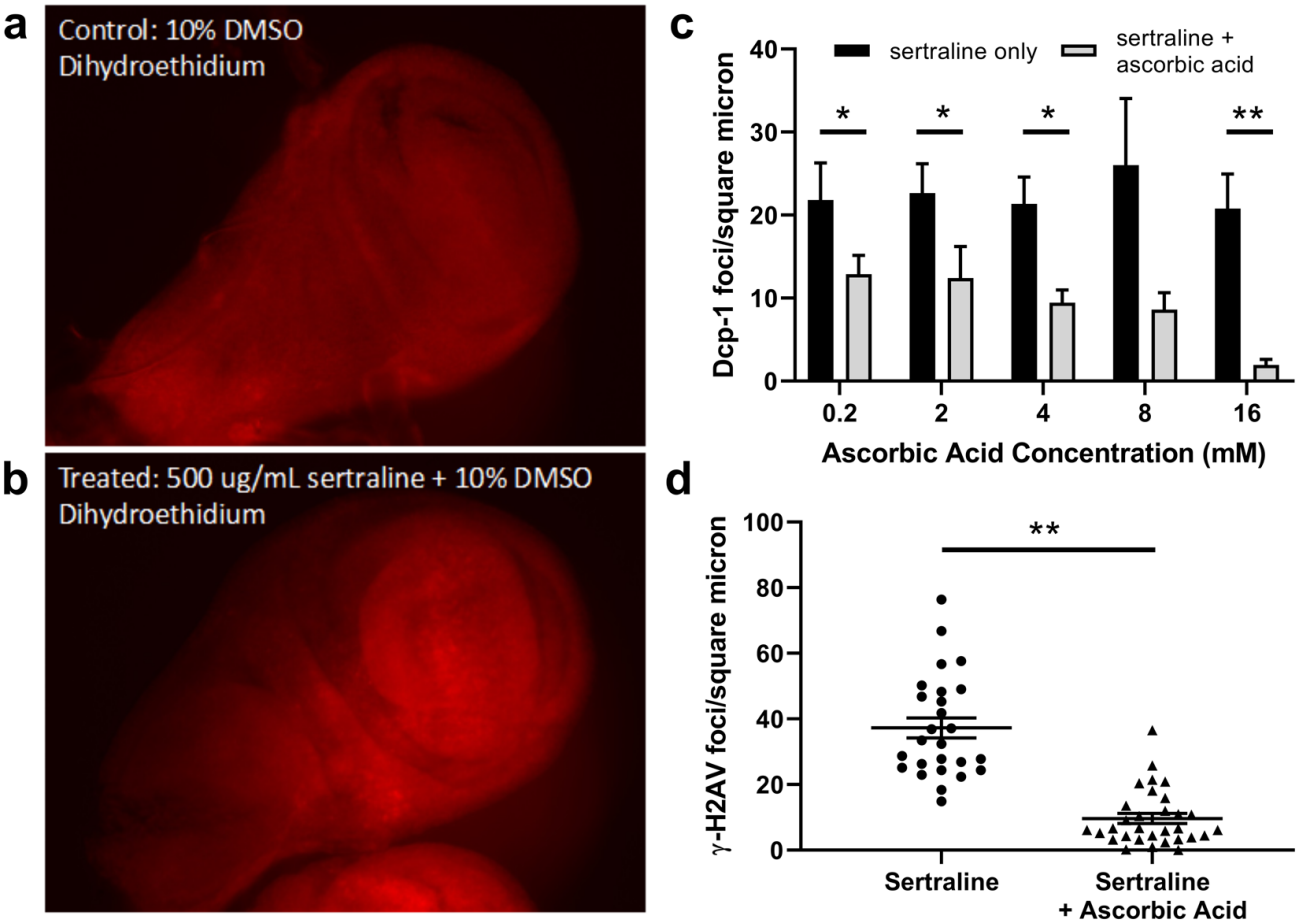
Addition of ascorbic acid ameliorates sertraline-induced increases in cell death and DNA damage. (**a** and **b**) Wing imaginal discs dissected from *SerT −/−* wandering third instar larvae were treated for 5 hours with DMSO or 500 μg/ml sertraline + DMSO and stained with dihydroethidium to indicate presence of superoxide ions. (**c**) Relative number of cleaved Dcp-1 foci per square micron in *SerT −/−* wing imaginal discs treated with 10 μg/ml sertraline plus increasing concentrations of ascorbic acid. Dcp-1 foci were counted and normalized to the area of each wing disc, measured from DAPI-stained images. For each trial, N ≥ 7 discs for sertraline-only groups and ≥8 discs for each concentration of ascorbic acid, error bars represent SEM. *p < 0.05, **p < 0.01 by ANOVA with Bonferroni correction. (**d**) Quantification of γ-H2Av foci per square micron for discs treated with 10 μg/ml sertraline or 10 μg/ml sertraline plus 8 mM ascorbic acid. N = 26 discs for sertraline and 31 discs for sertraline + ascorbic acid, error bars represent SEM. **p < 0.01 by unpaired t test with Welch’s correction.

To test whether sertraline might increase the amount of reactive oxygen species, we stained sertraline-treated *SerT* knockout wing imaginal discs with dihydroethidium (DHE). DHE is a compound that can diffuse through cell membranes and produces a red fluorescent ethidium product upon reaction with superoxide radicals^63^. Interestingly, we observed more overall DHE staining in sertraline-treated discs than in vehicle-treated controls (Fig. 5a,b), although the intensity of staining varied in different regions of each disc. Thus, sertraline may increase the concentration of superoxide radicals *in vivo*.

If sertraline’s mechanism of toxicity induction is indeed via oxidative damage, we reasoned that supplementation of an antioxidant might mitigate this toxicity and prevent cell death. To investigate this, we tested the ability of increasing concentrations of ascorbic acid, a known antioxidant, to affect the sertraline-mediated increase in apoptotic cell death observed in SerT knockout wing imaginal discs. Indeed, we observed that increasing concentrations of ascorbic acid resulted in decreases in cell death associated with sertraline treatment (Fig. 5c). Addition of 0.2, 4, 8, and 16 mM concentrations of ascorbic acid led to a 1.7-, 2.3-, 3-, and 10-fold decrease in Dcp-1foci/micron^2^, respectively, compared to sertraline-treated control discs (Fig. 5c). At a dose of 16 mM, almost no Dcp-1 foci were observed (1.9 foci/micron^2^, compared to 20.8 foci/micron^2^ in the sertraline only control).

To determine whether ascorbic acid could also modulate the increase in DNA double-strand breaks observed with sertraline, we employed immunostaining against γ-H2Av using SerT knockout wing imaginal discs treated with 10 μg/mL sertraline and 8 mM ascorbic acid. Strikingly, this concentration of ascorbic acid resulted in a 5.9-fold decrease in γ-H2Av foci (6.3 ± 4.9 foci/micron^2^ in sertraline-treated discs supplemented with ascorbic acid, compared to 37.5 ± 17.0 foci/micron^2^ with just sertraline) (Fig. 5d). Thus, antioxidant treatment is capable of ameliorating both the DNA damage and cell death observed with sertraline exposure.

### Antioxidant supplementation rescues delayed development and decreased survival of sertraline-treated larvae

**Figure 6:**
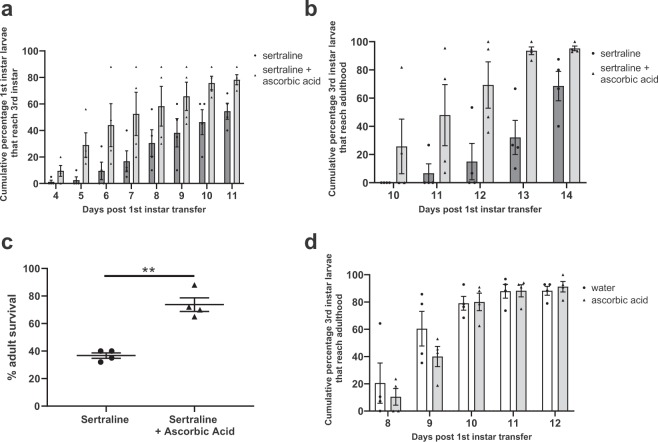
Addition of ascorbic acid rescues developmental and survival defects caused by larval exposure to sertraline. (**a**) Cumulative percentage of wandering third instar larvae following treatment of *SerT −/−* first instar larvae with 50 μg/ml of sertraline or 50 μg/ml sertraline + 8 mM ascorbic acid. N = 4 independent trials, error bars represent SEM. (**b**) Cumulative percentage of eclosed adults (number of adults/number of third instar larvae) following treatment of *SerT −/−* first instar larvae with 50 μg/ml sertraline or 50 μg/ml sertraline + 8 mM ascorbic acid. N = 4 independent trials, error bars represent SEM. (**c**) Total percentage of first instar larvae that survived to adulthood in the presence of 50 μg/ml sertraline or 50 μg/ml sertraline + 8 mM ascorbic acid. N = 4 independent trials, **p = 0.0025 by unpaired T test with Welch’s correction. (**d**) Cumulative percentage of eclosed adults (number of adults/number of third instar larvae) following treatment of SerT −/− first instar larvae with water or 8 mM ascorbic acid. N = 4 independent trials, error bars represent SEM.

Based on the immunostaining results, which show that the addition of ascorbic acid can largely abolish the negative genotoxic effects of sertraline, we performed development tracking assays to determine if the beneficial effects extend to the organismal level. First instar *SerT* knockout larvae were placed on food containing 50 μg/mL sertraline or 50 μg/mL sertraline + 8 mM ascorbic acid. Remarkably, the sertraline-mediated developmental delays were decreased when ascorbic acid was included in the food. Addition of ascorbic acid increased the rate of development of sertraline-treated larvae from first instar to third instar by 2-3 days (Fig. 6a) and from first instar to adulthood by a similar amount of time (Fig. 6b), although progression to these developmental stages was still slower than that of *SerT* knockout larvae not exposed to sertraline (compare Fig. 3 to Fig. 6). In addition, ascorbic acid significantly increased the percentage of sertraline-treated larvae that survived to adulthood (Fig. 6c). The effect of ascorbic acid on development was sertraline-specific, as inclusion of 8 mM ascorbic acid in food not containing sertraline did not significantly affect developmental rate from third instar larvae to adulthood (Fig. 6d). Overall, these data suggest that antioxidants can decrease sertraline-induced oxidative DNA damage in Drosophila, promoting regular development and survival.

## Discussion

### Sertraline induces DNA damage and cell death stochastically, mimicking its pattern of effects on human development

Because sertraline is one of the most commonly prescribed antidepressants^22^, a better understanding of the mechanistic basis behind the birth defects sporadically observed in children of mothers taking the drug is needed. Here, we utilized a Drosophila model system to determine the effects that sertraline may have on metazoan development. Our initial attempts to test the effects of feeding sertraline to females had no apparent impact, as we failed to see any obvious morphological defects in the embryos, larvae, or adult progeny derived from these females. Because most of the egg development and patterning in these females took place prior to sertraline exposure, this result is perhaps not surprising. Future work using quantitative morphometrics and transcriptional, proteomic, and epigenetic markers will be needed to delineate potential additional phenotypes that may not have been seen in our analysis.

We then turned to a larval feeding protocol, which exposes the developing organism to a physiologically relevant amount of sertraline during a critical stage of development. Although all larval tissues are likely exposed to the drug, we focused on imaginal discs as the target tissue. In Drosophila larvae, these discs contain rapidly dividing diploid cells that are preserved during metamorphosis and eventually form many of the external structures of the adult fly. When cultured *ex vivo*, cells within the discs continue to rapidly proliferate and maintain DNA damage checkpoints, making them a good model for early cell divisions in human embryos. The concentration of sertraline in the imaginal disc assays was within an order of magnitude of the estimated bioavailability of sertraline in humans^64^. Discs treated *ex vivo* with sertraline experienced high levels of oxidative stress, increased DNA damage, and heightened levels of apoptosis. Because these cellular processes are likely to be associated with mutagenesis, we anticipate that our findings may have implications for human developmental processes that occur in the presence of sertraline.

Notably, we observed that these effects are highly variable, paralleling the stochastic nature of sertraline-correlated birth defects in humans. For example, while the overall effect of sertraline ingestion by larvae was a general slowing of development, some sertraline-treated larvae developed at the same rate as untreated larvae. A possible explanation for this variability could be that larvae consumed different amounts of drug-containing food, resulting in variability in drug concentration between individuals. However, the amount of sertraline-induced DNA damage and apoptosis in wing imaginal discs also varied widely, with the distributions for both appearing to be almost bimodal (Fig. 4). The reasons for this are not known, but because the Drosophila in our experiments were genetically homogenous, the variability is likely due to stochastic differences in cellular responses to sertraline and not to genetic differences. This represents an exciting model system for future studies of variability and non-genetic heterogeneity, as physiological variability and stochasticity are now an important aspect of areas of developmental biology^65^ as well as cancer biology^7,66^.

Prior research has been mixed with regards to the genotoxic potential of sertraline. Pre-clinical trials did not find evidence for genotoxicity in mice, rats, rabbits, or dogs^11^ and a single human study that measured chromosome aberrations by cytogenetic staining showed no significant effect of sertraline^12^. In contrast, other investigations demonstrated that sertraline treatment induces apoptosis in human cancer cell lines^67^, inhibits cell proliferation, and decreases cellular viability^68,69^. The only other sertraline study in Drosophila showed mutagenicity at certain concentrations, but a dose-response effect was not observed^48^. Our study supports a model in which highly proliferative cells may be particularly sensitive to sertraline exposure, possibly including certain cell populations in a developing fetus.

### Partial rescue of sertraline-mediated effects by antioxidant supplementation

While a considerable literature on the risks associated with SSRIs exists, the debate has been largely about weighing risks to the mother (by avoiding SSRI use) vs. risks to the fetus (via exposure). Optimal care of both patient populations, as well as insight into molecular mechanisms, thus hinge on the identification of potential treatments that could reverse deleterious effects of SSRI therapies. To our knowledge, no prior studies have addressed the issue of how sertraline-induced damage could be prevented; we thus focused on validating one potential candidate approach to this problem, identified via the mechanistic information obtained in our study.

Based on the increased numbers of DNA double-strand breaks in sertraline-treated imaginal discs, we hypothesized that sertraline might induce oxidative damage, the repair of which could result in DNA single-strand breaks that are converted to double-strand breaks in replicating cells. We observed a decrease in DNA damage and apoptotic cell markers in sertraline-treated wing imaginal discs in the presence of the antioxidant ascorbic acid. In addition, larvae that ate food containing sertraline and ascorbic acid developed faster and had increased survival compared to larvae that ingested only sertraline. Together, these findings support a model that sertraline promotes genotoxicity through the induction of oxidative damage, which may have a greater (but not exclusive) effect on the viability of rapidly dividing cells.

It is important to note that while ascorbic acid decreased the cellular markers of sertraline-induced DNA damage to a level observed in larvae not exposed to sertraline, the developmental progression of larvae consuming sertraline and ascorbic acid was still slower than those of larvae not consuming sertraline. Thus, sertraline may have additional detrimental effects on cells, independent of its ability to induce DNA damage and possibly linked to an increased concentration of extracellular serotonin. Indeed, the observation that larvae lacking the serotonin reuptake channel develop slower and have slightly reduced survival compared to normal larvae (Fig. 3) supports this idea.

In conclusion, the results of our studies reveal an unanticipated genotoxic effect of sertraline that may partially explain the increased rate of birth defects in children of pregnant women taking the drug. They also suggest that potential negative effects of sertraline may be mitigated by endogenous antioxidant enzymes and/or supplementation with antioxidant compounds – a regime that does not utilize drugs with additional potential side effects, and thus is a very low-risk, likely high patient-compliance approach for optimizing fetal health.

## Methods

### Drosophila stocks and mutants

All experiments were carried out using stocks of *Drosophila melanogaster* that were maintained at 25 °C on a 12hr light:12hr dark cycle. *Oregon-R* (Bloomington stock number 25211) and *SerT* knockout (Bloomington stock number 36004) stocks were obtained from the Bloomington Drosophila Stock Center. The *SertT* knockout (*y*^1^ w^*^; Mi[MIC]SerT^*MI02578*^) has a Minos-Mediated Integration Cassette (MiMIC) transposon^70^, containing stop codons in all three reading frames, inserted into intron 3 of the *SerT* gene. RT-PCR was used to verify the lack of functional *SerT* transcript. Flies isogenic to the *SerT* knockout strain, but with an intact *SerT* gene, were created through excision of the *SerT*^*MI02578*^ transposon by crossing to flies carrying the Minos transposase (*w*^*1118*^*; noc*^*Sco*^*/SM6a, P{hs/MiT-2.4}*, Bloomington stock 24613). Precise excision of the transposon was verified by Sanger sequencing.

### Capillary feeder (CAFE) assays

Eight, 3-to-4-day-old, adult female flies were placed into a CAFE apparatus^49^ with capillary tubes containing either 5% sucrose or 5% sucrose + 250 μg/mL sertraline for 8 hours at 25 °C. Control CAFEs did not contain any flies. After 4 hours, capillary tubes were filled with additional solution. The volume of liquid lost due to evaporation in the control capillary tubes was calculated and subtracted from the volume of liquid lost in capillary tubes present in the fly-containing CAFEs. This allowed for calculation of the amount of liquid consumed by the flies, while also accounting for evaporation.

### Measurement of adult female fecundity and egg-hatching frequency

Cohorts of eight female flies were fed sucrose or sertraline using the CAFE assay^49^, then anesthetized using carbon dioxide gas and placed into cages capped with grape juice agar plates with equal numbers of male flies. Embryos were collected for 18 hours and the numbers of hatched and unhatched embryos were counted 48 hours after removal of the females. Hatching frequencies were calculated based on at least three trials. The first instar larvae that hatched from these eggs were then transferred to cornmeal medium so that they could be followed to adulthood to observe possible abnormalities. Statistical analysis for fecundity and hatching frequency was performed using two-tailed, unpaired t-tests.

### Preparation of food

Drosophila food was prepared by mixing Jazz-Mix (Fisher Scientific) with the manufacturer recommended amount of water and boiling for 5 minutes. Sertraline solution was prepared at concentrations of 50–500 µg/mL in 1% dimethyl sulfoxide (DMSO) solution and incubated at 55 °C until fully dissolved. For the larval feeding experiments, 500 µL of the solution was added to 4.5 mL of cooled (~55 °C) liquid food for final concentrations of 5–50 µg/mL sertraline. Control food was prepared by adding 500 μL of 1% DMSO solution without sertraline to 4.5 mL of cooled liquid food. For the antioxidant experiments, ascorbic acid was dissolved in water and added directly to the cooled liquid food.

### Developmental stage tracking

Flies were placed into collection cages with grape juice agar plates and yeast paste to collect embryos. The agar plates were removed after two hours and incubated at 25 °C overnight. 20–25 first instar larvae were individually transferred into vials containing the appropriate food solution within four hours of hatching. Transfers were done with a metal probe, being careful not to injure the larvae. These vials were kept at 25 °C, and the number of wandering third instar larvae (based on size and wandering behavior), pupae, and eclosed adults was tallied every 12–24 hours, depending on the experiment. Statistical analysis was performed using a one-way ANOVA with Bonferroni correction.

### Quantifying DNA damage and apoptosis

Wing imaginal discs were dissected from wandering third instar larvae^71,72^ and incubated in 100 µL of 0.7% NaCl solution containing 20% fetal bovine serum with either 0.02% dimethyl sulfoxide (DMSO) or 10 µg/mL sertraline in 0.02% DMSO for 5 hours at 25 °C. For measurement of ascorbic acid’s effects on DNA damage and apoptosis, 10 µg/mL sertraline solution was compared to 10 µg/mL sertraline solution containing increasing concentrations of ascorbic acid (0.2, 4, 8, and 16 mM) in separate trials. Treated discs were fixed with 1.48% formaldehyde for 30 minutes and incubated overnight with a 1:500 dilution of primary antibody anti-γ-H2Av (Rockland Inc.) or 1:100 dilution of Dcp-1 (Cell Signaling Technology). After 2 hours of incubation with secondary antibody solution (1:1000 goat anti-Rabbit IgG Rhodamine Red conjugated (Invitrogen) and 50 μg/mL DAPI in blocking solution), discs were mounted on microscope slides in 30 µL VECTASHIELD™ droplets and imaged with a Zeiss Z-stacking microscope using a 40X objective. 10–15 individual image slices from a Z-stack of the entire width of each wing disc were deconvolved using ZenPro image processing and stacked into one extended depth of field for each channel. A DsRed filter was used to visualize γ-H2Av or Dcp-1 foci, while a DAPI filter was used to visualize individual cell nuclei. ImageJ was used to quantify the number of γ-H2Av or Dcp-1 foci. Background in DsRed images was reduced until only foci were visible using intermodes auto-thresholding^73^, one of the most stringent means of histogram thresholding available, to ensure that background signal did not falsely inflate foci counts. All particles larger than 4 square microns were counted as foci. DAPI images were used to determine the area of each wing disc tested. γ-H2Av and Dcp-1 foci were normalized to wing disc area.

### Measurement of reactive oxygen species formation

The formation of reactive oxygen species was measured through dihydroethidium (DHE) staining. Sertraline solution was prepared at a concentration of 500 µg/mL in 10% DMSO and compared to 10% DMSO in picopure water as a negative control. Imaginal wing discs were dissected from third instar larvae and incubated in culture media containing each solution for 5 hours at 25 °C as described. Discs were incubated in 30 µM DHE solution (Thermo Fisher Scientific) for 6 mins protected from light, fixed in 7% formaldehyde for 5 minutes, washed with PBS, and immediately imaged using the RFP filter on a Zeiss Z-stacking microscope.

## Data Availability

The datasets generated during and/or analyzed during the current study are available from the corresponding author on reasonable request.
